# Feature Selection Based on Adaptive Particle Swarm Optimization with Leadership Learning

**DOI:** 10.1155/2022/1825341

**Published:** 2022-08-28

**Authors:** Zhiwei Ye, Yi Xu, Qiyi He, Mingwei Wang, Wanfang Bai, Hongwei Xiao

**Affiliations:** ^1^School of Computer Science, Hubei University of Technology, Wuhan 430070, China; ^2^Xining Big Data Service Administration, Xining 810000, China; ^3^Xining Zhiyun Digital Economy Research Institute, Xining 810000, China

## Abstract

With the rapid development of the Internet of Things (IoT), the curse of dimensionality becomes increasingly common. Feature selection (FS) is to eliminate irrelevant and redundant features in the datasets. Particle swarm optimization (PSO) is an efficient metaheuristic algorithm that has been successfully applied to obtain the optimal feature subset with essential information in an acceptable time. However, it is easy to fall into the local optima when dealing with high-dimensional datasets due to constant parameter values and insufficient population diversity. In the paper, an FS method is proposed by utilizing adaptive PSO with leadership learning (APSOLL). An adaptive updating strategy for parameters is used to replace the constant parameters, and the leadership learning strategy is utilized to provide valid population diversity. Experimental results on 10 UCI datasets show that APSOLL has better exploration and exploitation capabilities through comparison with PSO, grey wolf optimizer (GWO), Harris hawks optimization (HHO), flower pollination algorithm (FPA), salp swarm algorithm (SSA), linear PSO (LPSO), and hybrid PSO and differential evolution (HPSO-DE). Moreover, less than 8% of features in the original datasets are selected on average, and the feature subsets are more effective in most cases compared to those generated by 6 traditional FS methods (analysis of variance (ANOVA), Chi-Squared (CHI2), Pearson, Spearman, Kendall, and Mutual Information (MI)).

## 1. Introduction

Large amounts of data have been generated in various fields such as social media, healthcare, cybersecurity, and education in the past decades, and edge computing provides an effective solution for data storage and transmission. However, as the dimensionality of the data increases, the curse of dimensionality problem becomes common, which has a negative impact on the stability, security, and computational efficiency of edge computing. Feature selection (FS) is a data preprocessing technique in machine learning and data mining that has been applied to improve the performance of edge computing by eliminating irrelevant and redundant features in the datasets [1–3]. In general, it is a combinatorial optimization problem [4, 5] that tries to find the optimal feature subsets with essential information from the original datasets. Given a dataset with N features, there will be 2^N^ possible feature subsets, and the search space rises exponentially as the number of features increases [6, 7]. Hence, some traditional FS methods have received considerable interest due to their ability to evaluate feature importance and select a certain number of top-ranked features. These methods include statistical test (e.g., analysis of variance (ANOVA) [8, 9] and Chi-Squared (CHI2) [10, 11]), correlation criteria (e.g., Pearson [12], Spearman [13, 14], Kendall [15, 16]), and information theory (e.g., symmetrical uncertainty (SU) [17], mutual information (MI) [18, 19], and entropy [20]). However, the statistical test and correlation criteria techniques only consider the correlation between features and labels, and the feature subsets are not appropriate because some highly correlated but redundant features are selected. As a result, information theory techniques are applied to FS problems owing to their consideration of redundancy between features as well. Moreover, the redundancy calculation only focuses on the interaction between two features and fails to identify those of multiple features [21], which may ignore some important features. Therefore, how to find suitable feature subsets efficiently needs to be further investigated.

Metaheuristic algorithms such as monarch butterfly optimization (MBO) [22], slime mold algorithm (SMA) [23], moth search algorithm (MSA) [24], hunger games search (HGS) [25], hybrid rice optimization (HRO) [26], colony predation algorithm (CPA) [27], weighted mean of vectors (INFO) [28], grey wolf optimizer (GWO) [29], clonal flower pollination algorithm (FPA) [30], salp swarm algorithm (SSA) [31], Harris hawks optimization (HHO) [32], and particle swarm optimization (PSO), have been used to solve combinatorial optimization problems because of their dynamic exploration and exploitation capabilities in the search space, some of which have shown to be successful in FS problems [33, 34]. For instance, Shen and Zhang [29] proposed a two-stage GWO for processing biomedical datasets, which showed better performance in terms of time consumption and classification accuracy by removing more than 95.7% of the redundant features. Hussain et al. [32] developed an FS method based on HHO, which removed 87% of features and achieved 92% of classification accuracy. Yan et al. [30] presented a binary clonal FPA for some biomedical datasets, which enhanced population diversity and selected fewer features with strong robustness. Balakrishnan et al. [31] designed an FS method based on salp SSA, which increased the ability of particles to explore different regions by randomly updating their position and improved the confidence level by 0.1033% on 6 datasets. However, a series of parameters need to be set by users in these metaheuristic algorithms, and unsuitable parameters may lead to slow convergence and local stagnation. A lot of experiments and extensive experience are needed to find the appropriate parameter settings.

Compared with the above metaheuristic algorithms, PSO is applied to solve FS problem of its fast convergence and few parameters. However, the exploration and exploitation capabilities are influenced by parameter setting and population diversity as the number of features increases. Therefore, some improved PSO based on parameter updating and population diversity updating strategies have been proposed for FS. For example, Song et al. [35] developed a three-phase hybrid FS algorithm, which reduced the computational cost by using correlation-guided clustering and an improved integer PSO. Tran et al. [36] used a bare-bones PSO for FS, which reduced the search space of the problem and improve the search efficiency. Song et al. [37] also introduced a variable-size cooperative coevolutionary PSO for high-dimensional datasets, which divided a high-dimensional FS problem into multiple low-dimensional subproblems with a low computational cost. Hu et al. [38] presented a multi-objective PSO for FS, which achieved superior performances in approximation, diversity, and feature cost by introducing a tolerance coefficient. Hosseini Bamakan et al. [39] proposed a time-varying PSO-based FS method to deal with the network intrusion detection problem, which obtained a higher detection rate and lower false alarm rate by introducing a chaotic concept and time-varying parameters. Mafarja et al. [40] proposed a binary PSO-based FS method, which adopted a time-varying inertia weighting strategy and showed a superior convergence rate on some datasets. Huang et al. [41] utilized cut-point and feature discretization to expand the searching scope of PSO for gene expression datasets, which selected fewer features and maintained similar classification accuracy. Xue et al. [42] introduced adaptive parameters in PSO for high-dimensional datasets, which allowed particles to automatically adjust parameters during the search process and decreased time consumption. Moradi and Gholampour [43] used a PSO with the local search strategy for high-dimensional datasets, which adjusted the search process by considering the correlation information between distinct features. Chen et al. [44] introduced an FS method based on hybrid PSO and differential evolution (HPSO-DE), which enhanced population diversity by adopting mutation, crossover, and selection operators. Although the optimization ability of PSO is improved to some extent by the above techniques, the randomness of the search process may be increased and they lack consideration for jumping out of the local optima.

In the paper, an FS method based on adaptive PSO with leadership learning (APSOLL) is proposed, which combines parameter updating and population diversity updating strategies to compensate for the shortcomings of PSO. The adaptive updating strategy for parameters is used to guide particles to search in a more reasonable scope, and the leadership learning strategy is utilized to enhance population diversity. Overall, the main contributions of our work are as follows:

1. Based on the population state, an adaptive updating strategy for parameters is proposed to replace the constant parameters which guide particles to search in a more reasonable scope.
2. Adopting leadership learning strategies to provide valid population diversity by learning from the first three leaders in the population that enhances the exploration and exploitation capabilities of PSO.
3. The effectiveness of the proposed method is verified by comparing it with six traditional methods (ANOVA, CHI2, Pearson, Spearman, Kendall, and MI) and seven metaheuristic algorithms-based FS methods (GWO, HHO, FPA, SSA, LPSO, and HPSO-DE).

## 2. Background and Related Work

### 2.1. Overview of PSO

PSO is a population-based metaheuristic algorithm for simulating the predatory activities of bird and fish populations [45, 46], and each particle in the population has two properties: velocity vector *v*_*i*_ = (*v*_*i*1_, *v*_*i*2_, ⋯, *v*_*id*_) and position vector *x*_*i*_ = (*x*_*i*1_, *x*_*i*2_, ⋯, *x*_*id*_), where *d* denotes the dimension. In the search process of PSO, the velocity vectors are dynamically adjusted by the personal best position (*pbest*_*i*_) and the global best position (*gbest*) at the current stage, and the position vectors are the candidate solutions to the optimization problems, all of which are updated by equations (1)–(2).

(1) $$\begin{array}{c} v_{i}\left(t+1\right)=\omega \times v_{i}\left(t\right)+c_{1}r_{1}\left(p\;{\text{best}}_{i}-x_{i}\left(t\right)\right)+c_{2}r_{2}\left(g\text{ best}-x_{i}\left(t\right)\right), \end{array}$$

(2) $$\begin{array}{c} x_{i}\left(t+1\right)=x_{i}\left(t\right)+v_{i}\left(t+1\right). \end{array}$$

where *v*_*i*_ and *x*_*i*_ represent the velocity and position vectors of the *i*–*th* (*i* = 1, 2,…, N) particle, and the upper and lower limits of each dimension are set to 1 and 0, respectively. *ω* is defined as the inertia parameter, and it is a non-negative number. *c*_*1*_ and *c*_*2*_ are acceleration parameters, and the former represents the personal learning parameter and the latter represents the global learning parameter, which is used to control the search scope of particles and set by users. *r*_*1*_ and *r*_*2*_ are random numbers in [0, 1].

### 2.2. The Leadership Learning Strategy

**Figure 1:**
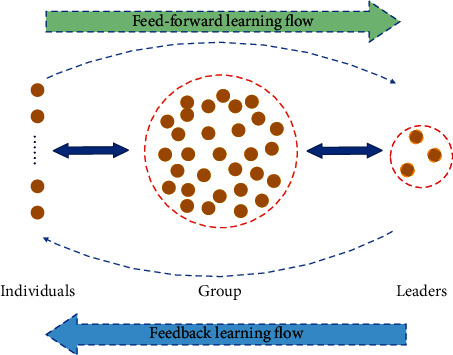
The framework of leadership learning.

Leadership learning strategy is a management concept that describes the dynamic process of feed-forward and feedback in a living system. Hirst et al. [47] suggested that learning activities of individuals will affect the decisions of leaders, and it is called feed-forward learning flow. Moreover, effective leaders may quickly identify key information in group development and have a lasting impact on the individuals and group activities through their decisions in turn, which is regarded as feedback learning flow. In the model of leadership learning strategy, feed-forward and feedback learning flow among individuals, groups, and leaders together determine the scope of the system development, and the framework is shown in Figure 1.

Based on the leadership learning strategy, GWO was proposed with effective exploration capability and acceptable time consumption by learning from the first three best solutions (leaders) of each iteration [48–51]. In the search process, the population is divided into four levels, sequentially *α*, *β*, *δ*, and *ω*, where *α*, *β*, and *δ* are regarded as leaders, the remaining particles *ω* are considered as individuals, and the population is considered group. Moreover, the particles and leaders learning from each other are considered as the leadership learning strategy, and it is shown in Equation (3).

(3) $$\begin{array}{c} \overset{⟶}{X_{1}}=\overset{⟶}{X_{\alpha }}-\overset{⟶}{A_{1}}\times \overset{⟶}{D_{\alpha }},\overset{⟶}{X_{2}}=\overset{⟶}{X_{\beta }}-\overset{⟶}{A_{2}}\times \overset{⟶}{D_{\beta }},\overset{⟶}{X_{3}}=\overset{⟶}{X_{\delta }}-\overset{⟶}{A_{3}}\times \overset{⟶}{D_{\delta }}. \end{array}$$

where $\overset{⟶}{X_{\alpha }}$, $\overset{⟶}{X_{\beta }}$, and $\overset{⟶}{X_{\delta }}$ are position vectors of *α*, *β*, and *δ*. $\overset{⟶}{D_{\alpha }}=\left|\overset{⟶}{C_{1}}\times \overset{⟶}{X_{\alpha }}-\overset{⟶}{X}\right|$, $\overset{⟶}{D_{\beta }}=\left|\overset{⟶}{C_{2}}\times \overset{⟶}{X_{\beta }}-\overset{⟶}{X}\right|$, $\overset{⟶}{D_{\delta }}=\left|\overset{⟶}{C_{3}}\times \overset{⟶}{X_{\delta }}-\overset{⟶}{X}\right|$ denote the distance between particles and leaders. $\overset{⟶}{C_{1}}$, $\overset{⟶}{C_{2}}$, and $\overset{⟶}{C_{3}}$ are random numbers from 0 to 2. The search scope of particles is controlled by the convergence factor $\overset{⟶}{A}$, which is computed as Equation (4).

(4) $$\begin{array}{c} \overset{⟶}{A}=2a\times r_{3}-a, \end{array}$$

where the variable *a* = 2(1 − *t*/*T*) is the control coefficient (T denotes the maximum number of iterations), and it decreases linearly from 2 to 0 during the search process.

## 3. The Proposed Method

In this section, an FS method based on APSOLL is presented to conduct classification on 10 UCI datasets. The corresponding techniques for the proposed method are described as follows:

### 3.1. Adaptive Updating Strategy for Parameters

During the search process of PSO, the search scope of particles is affected by convergence factor c_1_ and c_2_. In general, they are usually less than 2 and set to constant values by users [52–54]. However, the population is dynamically changed according to the optimal fitness value, it is appropriate to adaptively adjust c_1_ and c_2_ for better exploration and exploitation. Moreover, the change of fitness value during the iteration reflects the state of the population, thus the adaptive updating strategy is proposed based on this case, and it is used to replace the convergence factor, which is shown in equations (5)‒(6).

(5) $$\begin{array}{c} m=\left\{\begin{array}{cc} m+1, & \text{ if fitness}\left(t\right)=\text{fitness}\left(t|-|1\right),\\ 0, & \text{otherwise,} \end{array}\right. \end{array}$$

(6) $$\begin{array}{c} c={\left(\frac{m}{T}\right)}^{2/3}+1, \end{array}$$

where *m* is a variable and initially set to 0, and it is increased by 1 if the fitness value is improved in the next iteration, otherwise the value of which is always 0. Thus, *c* is dynamically changed between 1 and 2 during the search process, and it is gradually increased if the algorithm falls into the local optima.

### 3.2. The Search Process of Leadership Learning Strategy

The population diversity of PSO may be inadequate due to the strategy learned from *pbest_i_* and *gbest*. Smith [55] proposed that the more leaders of individuals engage feed-forward and feedback in a living system, the more possible it is for the group to change, innovate, and cooperate. However, the time consumption will increase as the number of leaders increases during the process. Therefore, inspired by GWO, the leadership learning strategy from 3 leaders is used to reconstruct the velocity vectors of PSO, which will increase population diversity and provide more accurate information for better exploration and exploitation. In addition, an adaptive parameter *c* is combined to guide the particles to search in a more reasonable scope, and the process is shown in Equation (7).

(7) $$\begin{array}{c} v_{i}\left(t+1\right)=\omega \Delta v_{i}\left(t\right)+\frac{c}{2}\times r_{4}\left(\overset{⟶}{X_{1}}-x_{i}\left(t\right)\right)+\frac{c}{3}\times r_{4}\left(\overset{⟶}{X_{2}}-x_{i}\left(t\right)\right)+\frac{c}{4}\times r_{4}\left(\overset{⟶}{X_{3}}-x_{i}\left(t\right)\right), \end{array}$$

where $\overset{⟶}{X_{1}}$, $\overset{⟶}{X_{2}}$ and $\overset{⟶}{X_{3}}$ represent the leadership learning strategy. *r*_4_ is a random number between 0 and 1. c is updated by (6), it is dynamically changed between 1 and 2 during the search process, and it is gradually increased if the algorithm falls into the local optima. The cooperation of *c*/2, *c*/3 and *c*/4 will allow particles to search in a more reasonable scope with higher possibilities.

As for the leadership learning strategy, Hu et al. [50] proposed that the convergence factor $\left|\overset{⟶}{A}\right|$ greater than 1 shows better exploration capability and less than 1 shows better exploitation capability. However, it can be seen from (4) that $\left|\overset{⟶}{A}\right|$ is linearly decreased and always less than 1in the last 50% of iterations, and the exploration capability is insufficient when the algorithm is trapped in the local optima in this case. Hence, it is considered to increase the possibility that $\left|\overset{⟶}{A}\right|$ is greater than 1 at this stage and it is modified as shown in Equation (8).

(8) $$\begin{array}{c} \overset{⟶}{A}=2^{c}a\times r_{5}-a. \end{array}$$

where *r*_5_ is a random number in [0, 1], and $\left|\overset{⟶}{A}\right|$ is adaptively changed during the search process. It will be greater than 1 with a higher possibility and thus enhance the exploration capability when the algorithm falls into the local optima.

### 3.3. The Encoding Schema

The core object of the proposed method is to select a suitable expression form for FS and establish a reasonable mapping between the solutions and the feature subsets. The candidate solutions that are binarized are used to represent the features, where “1” denotes the feature is selected and “0” illustrates the feature is abandoned. For instance, there is a feature dataset with 10 features, and the candidate solution is coded as 1010000011, which means the 1st, 3rd, 9th, and 10th features are selected and the others are abandoned. The position vector of each particle is binarized according to Equation (9).

(9) $$\begin{array}{c} Xb_{id}=\left\{\begin{array}{cc} 1, & \text{ if }x_{id}>0.5,\\ 0, & \text{otherwise,} \end{array}\right. \end{array}$$

where *Xb*_*i*_=(*Xb*_*i*1_, *Xb*_*i*2_, ⋯ , *Xb*_*id*_), *i* and *d* denote the number of particles and the number of features, respectively.

### 3.4. The Definition of Objective Function

The feature subsets generated by FS methods for classification have two main goals, which are maximizing the classification accuracy (minimizing the classification error) and minimizing the number of selected features. As a mainstream classifier, K nearest neighbor (KNN) [56–58] is utilized for FS due to its advantages of simplicity and insensitivity to noisy data. Furthermore, how to reduce the number of selected features is considered another core issue. The ultimate goal is to obtain the optimal feature subsets with essential information from the original datasets while achieving higher classification accuracy with fewer features. Hence, the objective function that combines the classification accuracy and the number of selected features is adopted and it is defined as Equation (10).

(10) $$\begin{array}{c} \text{Fitness}\left(X\right)=\theta \times \text{acc}\left(X\right)+\left(1-\theta \right)\times \left(1-\frac{\#X}{N}\right). \end{array}$$

where acc (*X*) denotes the classification accuracy of the feature subsets, #*X* and *N* represent the number of features in the feature subset and the original dataset. *θ* is a weighting factor to balance the classification accuracy and the number of selected features, and it is set to 0.7.

### 3.5. Implementation of the Proposed Method

**Figure 2:**
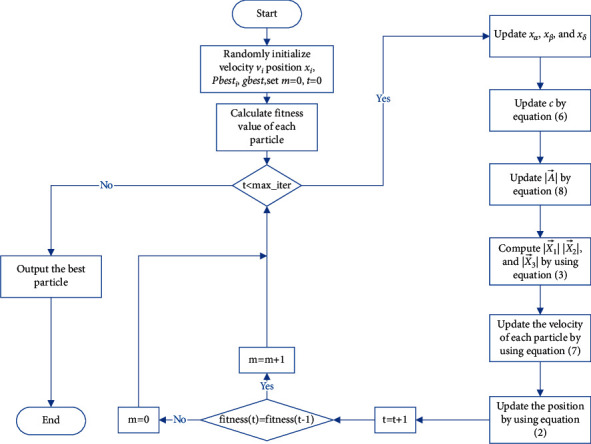
The flowchart of APSOLL.

The main process of APSOLL is to search for the optimal feature subsets with essential information from the original datasets and apply it for classification, and the pseudocode is shown in Algorithm 1. Among these, the particles are binarized to determine the corresponding feature subsets in each iteration, and the leaders are determined by computing the fitness function, which is used to guide the search process.  Figure 2 shows the flowchart of APSOLL. When the algorithm starts running, it randomly initializes the velocity vector *v*_*i*_, position vector *x*_*i*_, *pbest*_*i*_, *gbest*, and sets *m* *=* *0* and *t* *=* *0*. In each iteration, the fitness value of each particle is calculated in order to find the optimal three solutions (leaders). Based on the information provided by the leader, the velocity of the particles and the position of the population are updated. In this process, if the optimal fitness value is not changed, the adaptive parameter *m* is added by 1. The algorithm run is ended and the optimal solution is binarized when the maximum number of iterations is reached.

## 4. Experimental Design

All experimental procedures are implemented using *Python* 3.8 in a PC with Intel(R) Core (TM) i5-9400 @ 2.9 GHz CPU, and 16 GB DDR4 of RAM under Windows 10 Operating System. 10 public datasets are used to assess the quality of the proposed method. APSOLL is compared with 7 metaheuristic algorithms to evaluate the optimization ability, and 6 traditional FS methods such as ANOVA, CHI2, Pearson, Spearman, Kendall, and MI are used to analyze the effectiveness of the feature subsets selected by the proposed method.

### 4.1. Datasets Description

10 datasets from the UCI machine learning database are used to evaluate the performance of the proposed method, including myocardial infarction complications (MIC), urban, SCADI, arrhythmia, madelon, isolet5, multiple features (MF), Parkinson's disease (PD), CNAE-9, and QSAR, all of which have more than 100 features, with the number of classes ranging from 2 to 26 and instances ranging from 69 to 2600, and the details of datasets are shown in Table 1. In the experiments, each dataset is randomly divided into two parts: a total of 70% of the instances are chosen as the training data, and the remaining 30% are used as the testing data. Li et al. [54]described in detail why the dataset dividing approach was adopted.

### 4.2. Parameters Setting for Metaheuristic Algorithms

As for APSOLL, the search process requires only one inertia weight parameter *ω* to be set. In addition, some commonly used FS methods based on metaheuristic algorithms are adopted to evaluate the optimization ability, such as GWO, PSO, HHO, FPA, SSA, LPSO, and HPSO-DE. Among them, LPSO [40] and HPSO-DE [44] are classical benchmark PSO-based FS methods by adopting parameter updating and population diversity updating strategies, respectively. The parameters of each metaheuristic algorithm are set based on the published literature, which is shown in Table 2. Furthermore, the binary encoding scheme is utilized for each metaheuristic algorithm and it is run independently 30 times to take the average as the result in order to eliminate the influence of randomness.

## 5. Results and Discussion

### 5.1. Experimental Results of Different Metaheuristic Algorithms

**Figure 3:**
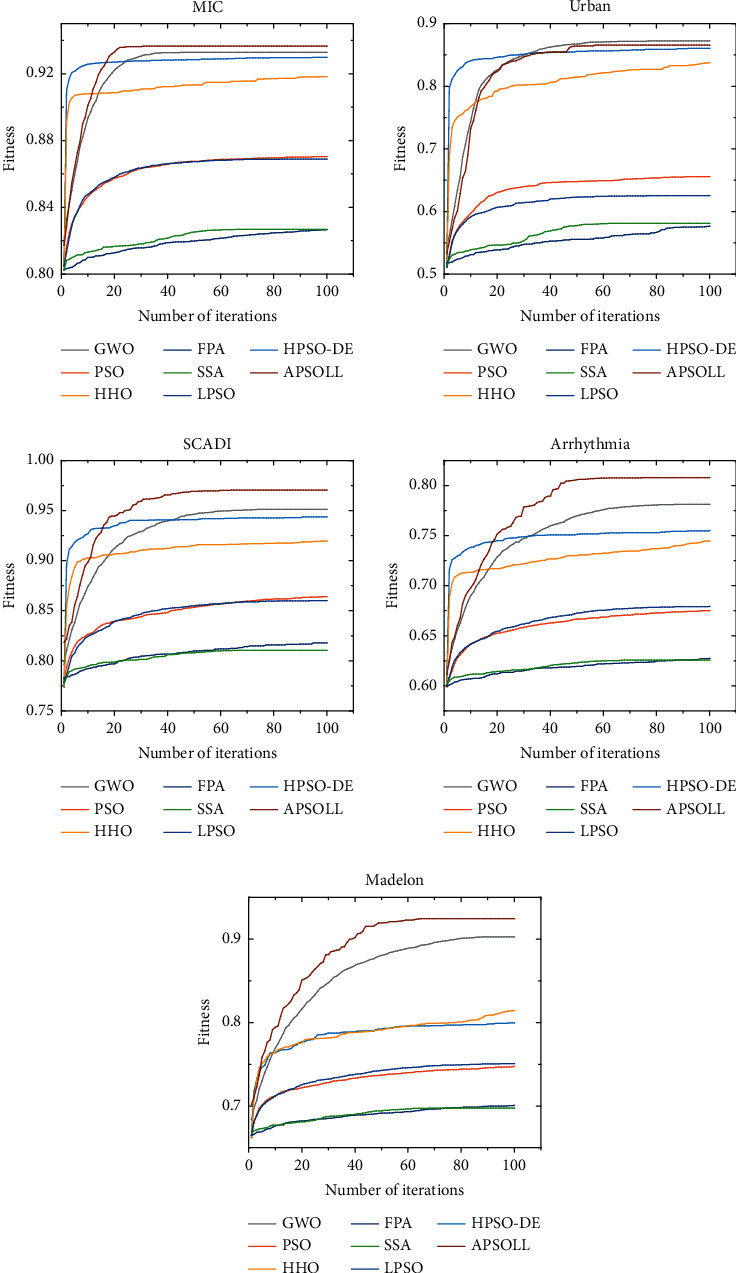
The average convergence curves of different metaheuristic algorithms for datasets below 500 dimensions.

**Figure 4:**
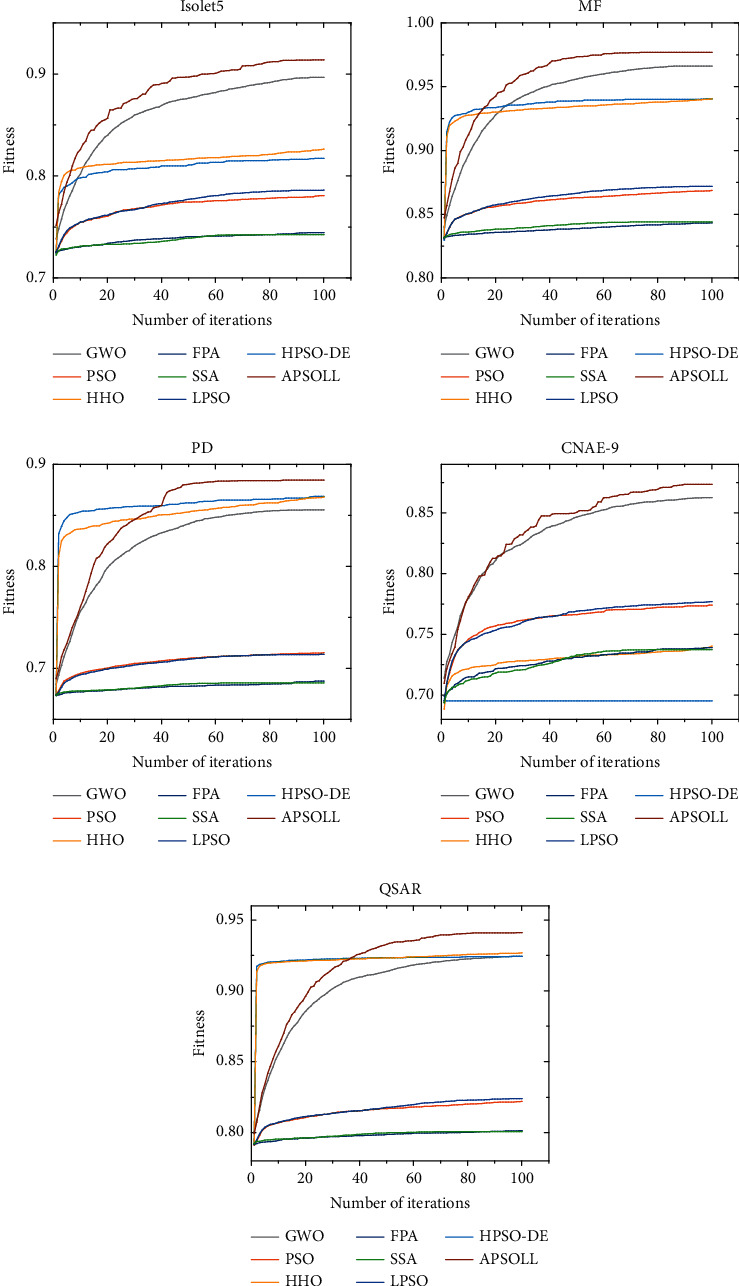
The average convergence curves of different metaheuristic algorithms for datasets above 500 dimensions.

**Figure 5:**
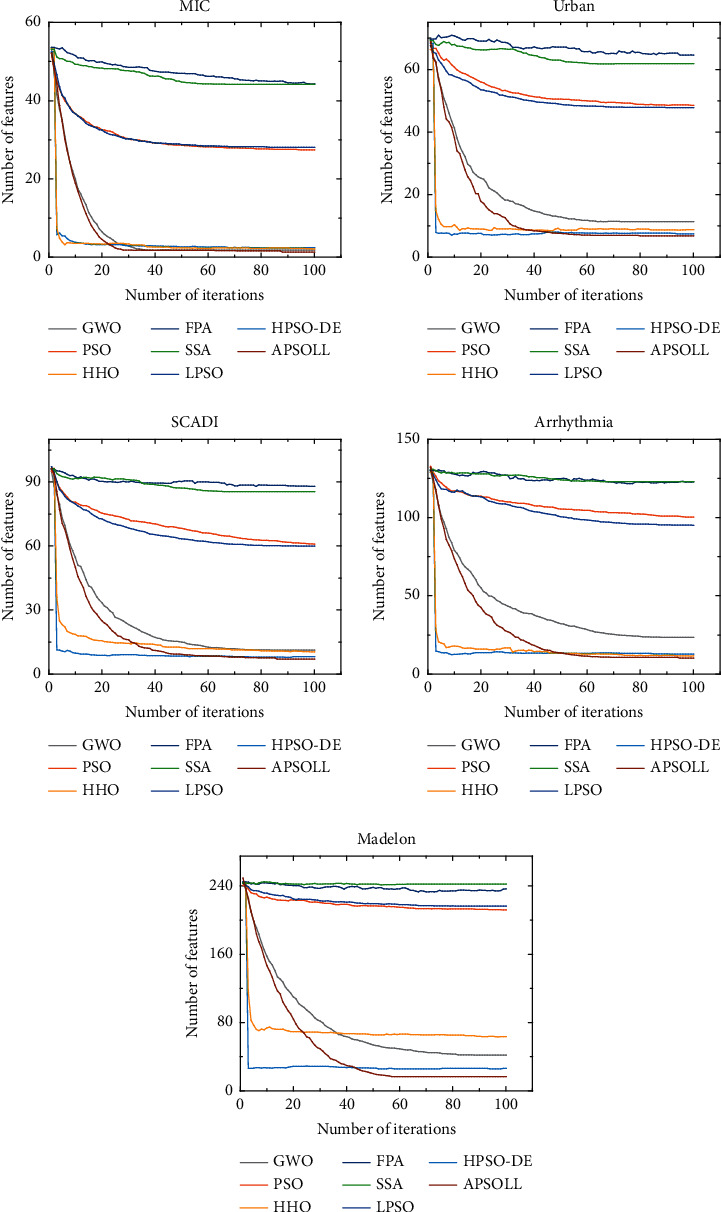
The average number of selected features for datasets below 500 dimensions by different FS methods based on metaheuristic algorithms.

**Figure 6:**
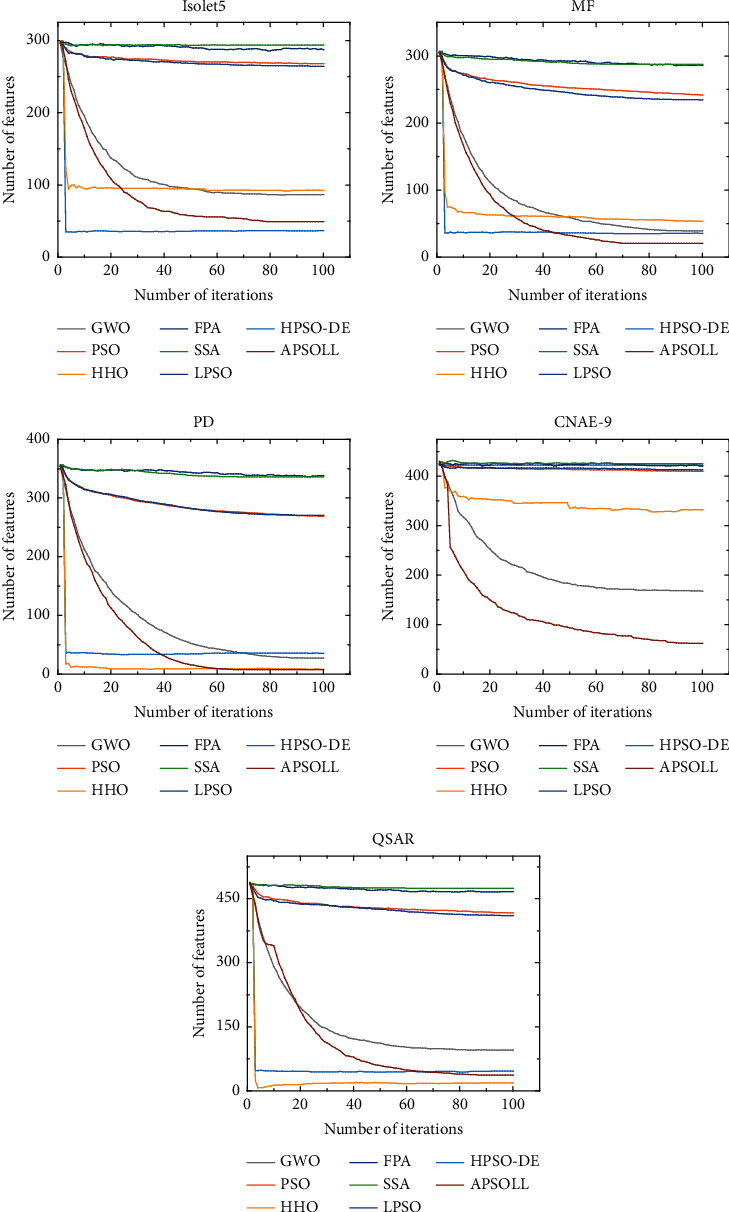
The average number of selected features for datasets above 500 dimensions by different FS methods based on metaheuristic algorithms.

The optimization ability of APSOLL is evaluated from the fitness value, classification accuracy, number of selected features, and CPU time. The average convergence curves of the fitness value are shown in Figures 3-4, and the number of selected features in the search process is shown in Figures 5-6. In the experiment, the *t*-test with a significance level of 0.05 is used to determine whether the results obtained from the proposed algorithm are statistically significantly different from other metaheuristic algorithms, and the experimental results are presented in Tables 3-4, where *Fit*, *Acc*, and *#F* denote the fitness values, classification accuracy and number of selected features after 30 independent runs, and *Time* presents the CPU time of the whole process (in seconds). *S*_*fit*_, *S*_*acc*_, and *S*_*f*_ display the *t*-test results, where “+” or “−” means the result is worse or better than the proposed method and “=” means they are similar in the *t*-test. In other words, the more “+”, the better the proposed methods.

From the variation curves of the fitness value, it is shown that APSOLL has achieved better fitness values on all datasets, which means the optimization ability of APSOLL is better than other metaheuristic algorithms by adopting the adaptive updating and leadership learning strategy. From Figures 3–4, it can be observed that HHO and HPSO-DE converge prematurely on most datasets, and PSO, SSA, FPA, and LPSO converge slower and have poor overall performance. In contrast, APSOLL achieves a balance in convergence speed and performance. In terms of classification accuracy, APSOLL-based FS method exceeds 80% on average in 9 of the 10 datasets, especially on MF, which has reached 98.07%. As it can be seen in Figures 5–6, PSO, SSA, FPA, and LPSO have limited performance in reducing the size of feature subsets, while APSO performs better than other methods on most datasets during the iterative process. In Tables 3–4, the number of selected features by APSOLL is less than those of other metaheuristic algorithms in most cases. A total of 30%–50% of features in the original datasets are selected by FPA and SSA, while less than 8% of features are selected by APSOLL. In particular, only 7.58 features are selected on average from the original 754 features on PD. As for CPU time, APSOLL consumes less time on MIC and madelon compared to other metaheuristic algorithms. Moreover, although it consumes slightly more time on other datasets, it performs better in the two main aims of the classification accuracy and the number of selected features.

In summary, the optimization ability of APSOLL is better than other metaheuristic algorithms, and the suitable feature subsets are selected with higher classification accuracy and fewer features at an acceptable time.

### 5.2. Experimental Results of Traditional Methods

**Figure 7:**
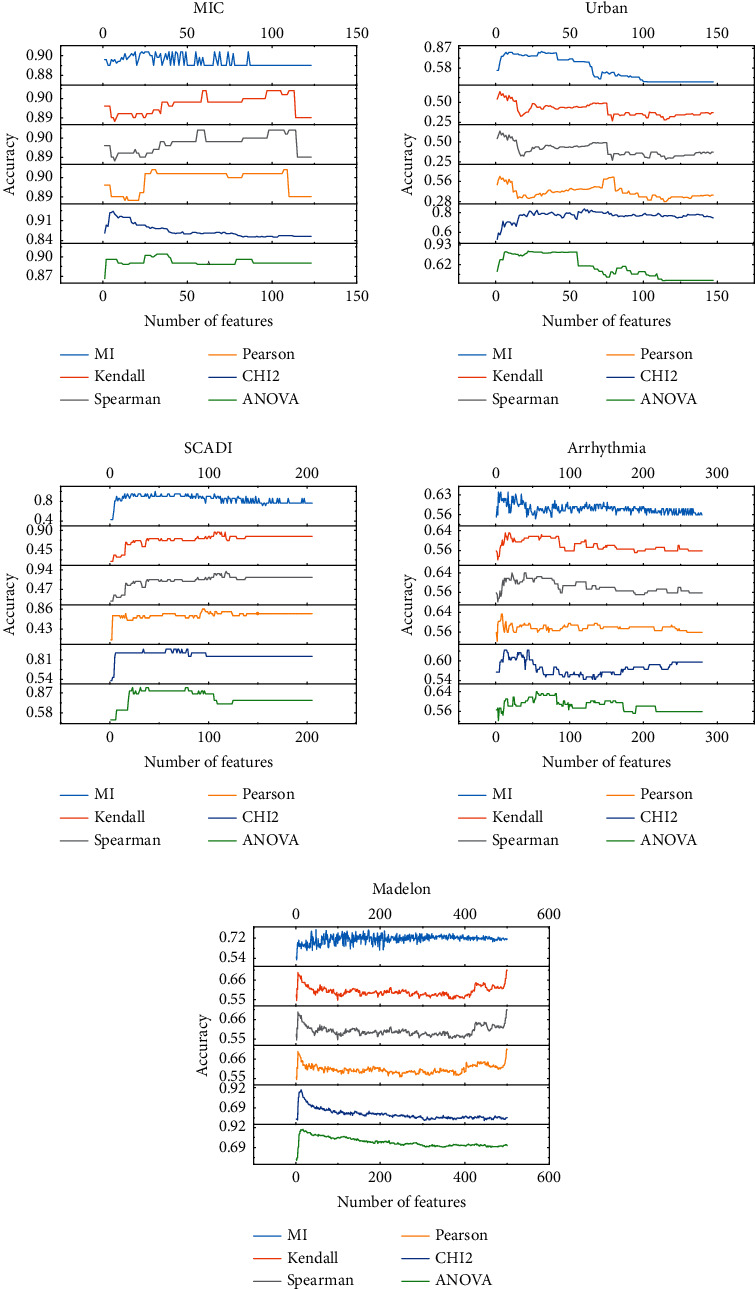
The classification accuracy of 6 traditional FS methods in selecting different numbers of features for datasets below 500 dimensions.

**Figure 8:**
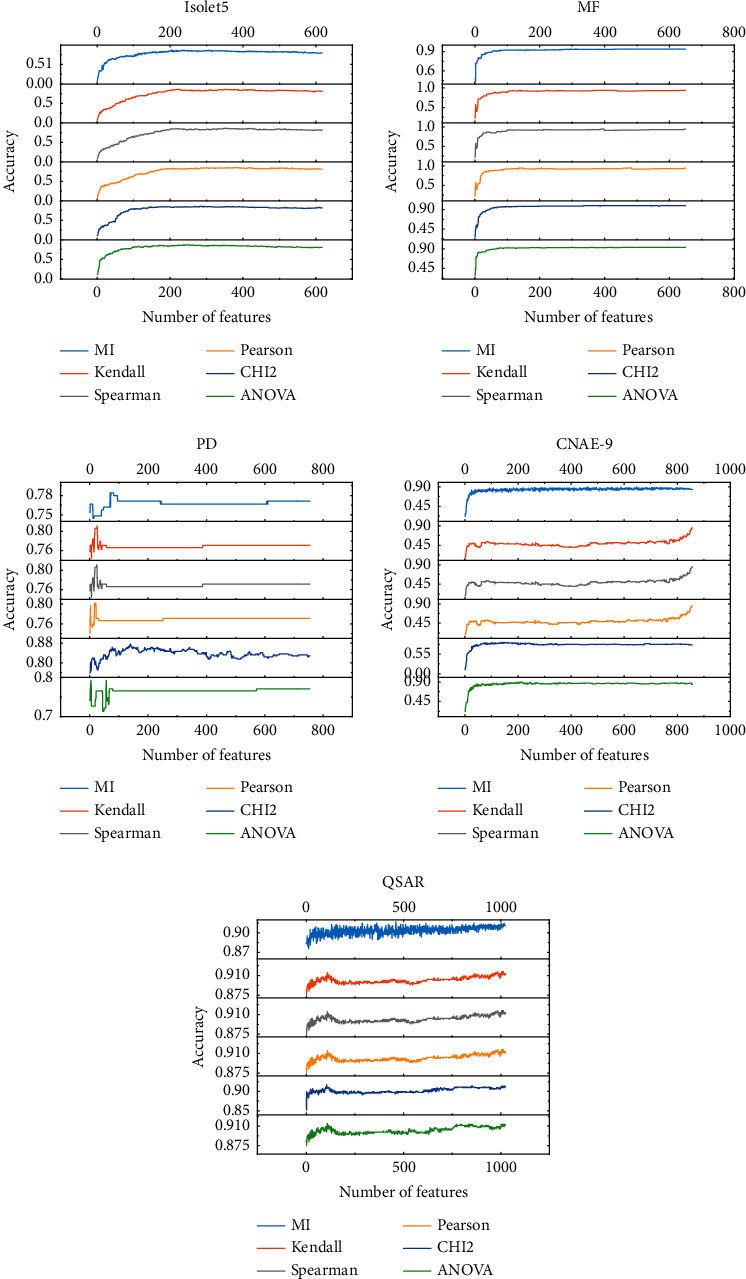
The classification accuracy of 6 traditional FS methods in selecting different numbers of features for datasets above 500 dimensions.

To demonstrate the effectiveness of APSOLL-based FS method, the performance is compared with that of 6 traditional methods. Figures 7‒8 show the classification accuracy of 6 traditional FS methods for different numbers of selected features, and the optimal solutions of the proposed and traditional methods are presented in Table 5.

It is observed from Figures 7‒8 that the traditional methods are difficult to improve the classification accuracy by sequentially increasing the number of features when a certain level is reached. In comparison, more suitable feature subsets are obtained by the metaheuristic algorithm-based FS method, among these, APSOLL has better performance. In addition, it is not the case that the more features selected, the higher the classification accuracy is, which indicates that the redundancy among features affects the classification performance on most datasets.

As can be seen from Table 5, it is clear that the classification accuracy is improved by at least 1.28% on average via the proposed method on 5 datasets, especially on arrhythmia and isolet5, with 11.77% and 4.26%, respectively. Although the classification accuracy of the proposed method is about 2% on average lower than traditional methods on myocardial, MF, PD, and CNAE-9, the number of selected features is lower than that of these methods, only 2, 21, 9, and 64 features are selected, respectively. To further analyze the number of selected features, fewer features are selected by the proposed method on 6 datasets. Among them, it is noticed that more than 30% of the features are selected by traditional methods on Isolet5 and MF, while only 7.46% and 3.24% of the features are selected by the proposed method, respectively. In terms of time consumption, traditional methods are affected by the number of features due to the sequential addition of features to the feature subsets, and its time consumption increase dramatically as the number of features increases, while APSOLL performs more stability on most datasets because its dynamic exploration and exploitation capabilities, and the CPU time is still acceptable. In brief, the proposed method is dependable and effective for solving FS problems compared with traditional methods.

## 6. Conclusions and Future Work

In the paper, APSOLL is proposed for FS, which enhances exploration and exploitation capabilities by utilizing an adaptive updating strategy to guide the population search in a more reasonable scope and the leadership learning strategy to increase population diversity. Experimental results in comparison with other FS methods based on metaheuristic algorithms reveal that APSOLL offers better optimization ability and selects the suitable feature subsets within an acceptable time. Moreover, APSOLL-based FS method achieves better or approximate classification accuracy by selecting less than 8% of features from the original datasets compared to other traditional methods. In conclusion, the suitable feature subsets are selected by the proposed method while ensuring a proper balance between the classification accuracy and the number of selected features. In the future, it is interesting to decrease the CPU time of APSOLL by combining the feature ranking and applying it to process ultrahigh dimensional datasets.

## Figures and Tables

**Algorithm 1:**
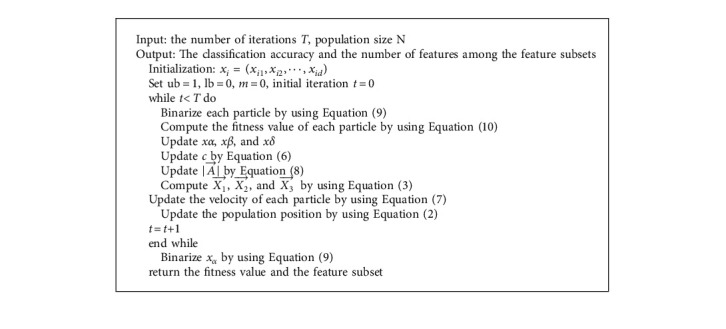
: FS based on APSOLL.

**Table 1:** Details of datasets.

Dataset	Number of features	Number of instances	Number of classes
MIC	124	1700	7
Urban	147	507	9
SCADI	205	69	6
Arrhythmia	279	452	13
Madelon	500	2600	2
Isolet5	617	1559	26
MF	649	2000	10
PD	754	756	2
CNAE-9	857	1080	9
QSAR	1024	1687	2

**Table 2:** Parameters Setting of different metaheuristic algorithms.

Algorithms	Parameters	Values
Common settings	Number of iterations	*T* *=* *100*
	Population size	*N* *=* *30*
	The upper limit of particle position	*ub* *=* *1*
	The lower limit of particle position	lb *=* *0*

GWO	Correlation coefficient	*a* decreases linearly from 2 to 0

PSO	Acceleration factor	*c*_*1*_ = 2, *c*_*2*_ = 2
	Inertia weight	*w* = 0.9

HHO	Levy component	*β* = 0.8

FPA	Acceleration factor	*c*_*1*_ = 2, *c*_*2*_ = 2
	Levy component	*β* = 1.5
	Switch probability	*P*=0.8

SSA	Convergence factor	*C* decreases linearly from 2 to 0

LPSO	Acceleration factor	*c*_*1*_ = 2, *c*_*2*_ = 2
	Upper limit of inertia weight	*wmax* = 0.9
	Lower limit of inertia weight	*wmin* = 0.4

HPSO-DE	Acceleration factor	*c*_*1*_ = 2, *c*_*2*_ = 2
	Crossover rate	*CR* = 0.2
	Scaling factor	*F* = 0.5
	Predefined generation	*G* = 5
	Inertia weight	*w* = 0.9

APSOLL	Inertia weight	*w* = 0.9

**Table 3:** Comparisons between APSOLL and other metaheuristic algorithms for datasets below 500 dimensions.

Datasets	Method	Fit (std.)	S_fit_	Acc (std.)	S_acc_	#F (std.)	*S *_f_	Time
MIC	GWO	93.28 (0.27)	+	91.03 (0.40)	+	1.80 (0.65)	=	125.37
	PSO	87.04 (0.96)	+	91.03 (0.48)	+	27.40 (3.49)	+	220.42
	HHO	91.83 (1.76)	+	89.08 (2.60)	+	2.13 (2.42)	=	162.52
	FPA	82.66 (0.53)	+	90.63 (0.75)	+	44.2 (2.50)	+	217.76
	SSA	82.68 (0.87)	+	90.65 (0.78)	+	44.2 (3.23)	+	122.10
	LPSO	86.89 (0.89)	+	91.08 (0.54)	+	28.13 (3.48)	+	220.95
	HPSO-DE	92.99 (0.38)	+	90.83 (0.45)	+	2.43 (1.09)	+	133.59
	APSOLL	93.65 (0.35)	*∗*	91.40 (0.56)	*∗*	1.33 (0.47)	*∗*	122.10

Urban	GWO	87.21 (4.12)	=	85.03 (16.03)	=	11.33 (2.70)	+	96.00
	PSO	65.57 (5.57)	+	64.97 (13.90)	+	48.53 (5.89)	+	160.65
	HHO	83.78 (3.18)	+	79.43 (14.26)	+	8.93 (5.41)	=	94.06
	FPA	57.64 (2.87)	+	58.26 (10.28)	+	64.40 (6.52)	+	163.10
	SSA	58.10 (3.92)	+	58.17 (10.39)	+	61.83 (5.88)	+	162.84
	LPSO	62.53 (4.90)	+	60.41 (11.97)	+	47.80 (5.17)	+	163.89
	HPSO-DE	86.06 (1.20)	=	82.24 (14.30)	=	7.40 (2.11)	=	47.19
	APSOLL	86.60 (2.18)	*∗*	82.84 (20.46)	*∗*	6.83 (1.91)	*∗*	75.32

SCADI	GWO	95.13 (2.04)	=	95.40 (2.88)	=	11.23 (7.49)	=	29.19
	PSO	86.43 (3.22)	+	93.33 (4.19)	+	60.87 (8.10)	+	124.32
	HHO	91.95 (3.61)	+	90.63 (4.51)	+	10.23 (7.14)	=	24.98
	FPA	81.80 (3.48)	+	92.38 (4.19)	+	87.90 (7.17)	+	147.73
	SSA	81.05 (3.59)	+	90.79 (4.59)	+	85.47 (8.11)	+	152.42
	LPSO	86.01 (3.12)	+	92.54 (4.20)	+	59.90 (6.65)	+	100.95
	HPSO-DE	94.38 (2.38)	+	93.65 (3.33)	+	8.07 (2.89)	=	23.31
	APSOLL	97.04 (1.63)	*∗*	97.22 (2.35)	*∗*	6.92 (2.75)	*∗*	33.66

Arrhythmia	GWO	78.11 (1.31)	+	72.33 (1.70)	+	23.48 (5.65)	+	161.93
	PSO	67.50 (1.28)	+	68.97 (1.98)	+	100.23 (8.12)	+	164.50
	HHO	74.48 (1.94)	+	65.29 (3.20)	+	11.40 (10.72)	=	127.69
	FPA	62.73 (1.07)	+	65.59 (1.97)	+	122.57 (7.68)	+	160.16
	SSA	62.56 (1.30)	+	65.39 (1.77)	+	122.93 (6.44)	+	159.65
	LPSO	67.92 (1.39)	+	68.77 (1.80)	+	95.03 (6.31)	+	167.47
	HPSO-DE	75.49 (0.86)	+	66.96 (1.39)	+	12.87 (2.50)	=	80.80
	APSOLL	80.82 (1.45)	*∗*	74.14 (1.75)	*∗*	10.08 (3.95)	*∗*	113.36

Madelon	GWO	90.28 (1.00)	+	89.71 (1.17)	+	42.00 (7.01)	+	310.38
	PSO	74.72 (1.12)	+	82.04 (1.18)	+	211.73 (12.36)	+	327.80
	HHO	81.44 (3.82)	+	78.95 (3.48)	+	216.47 (10.49)	+	399.71
	FPA	75.08 (0.86)	+	77.52 (1.16)	+	236.67 (9.62)	+	320.63
	SSA	70.06 (1.21)	+	77.53 (1.57)	+	242.17 (8.97)	+	322.84
	LPSO	75.07 (1.18)	+	82.94 (1.58)	+	63.70 (37.22)	+	325.15
	HPSO-DE	79.98 (1.72)	+	73.64 (2.56)	+	26.13 (5.06)	+	301.25
	APSOLL	92.44 (0.44)	*∗*	90.65 (0.62)	*∗*	16.92 (4.75)	*∗*	259.51

**Table 4:** Comparisons between APSOLL and other metaheuristic algorithms for datasets above 500 dimensions.

Datasets	Method	Fit (std.)	S_fit_	Acc (std.)	S_acc_	#F (Std.)	S_f_	Time
Isolet5	GWO	89.66 (1.01)	+	91.23 (1.38)	=	86.53 (9.14)	+	212.36
	PSO	78.10 (1.04)	+	87.31 (1.44)	+	268.07 (10.71)	+	219.31
	HHO	82.61 (1.71)	+	81.60 (1.83)	+	92.57 (26.83)	+	283.60
	FPA	74.45 (0.89)	+	83.50 (1.34)	+	287.73 (10.48)	+	211.13
	SSA	74.25 (1.02)	+	83.60 (1.45)	+	293.53 (8.69)	+	207.08
	LPSO	78.61 (0.98)	+	87.79 (1.40)	+	264.42 (10.19)	+	215.30
	HPSO-DE	81.72 (1.08)	+	76.42 (1.68)	+	36.53 (5.12)	−	215.85
	APSOLL	91.37 (0.49)	*∗*	91.08 (0.55)	*∗*	48.92 (2.36)	*∗*	219.14

MF	GWO	96.63 (0.54)	+	97.77 (0.54)	=	39.27 (6.89)	+	225.82
	PSO	86.86 (0.72)	+	97.19 (0.54)	+	241.73 (13.51)	+	274.84
	HHO	94.04 (0.95)	+	94.98 (0.98)	+	52.93 (13.66)	+	303.36
	FPA	84.31 (0.53)	+	96.47 (0.60)	+	286.13 (6.77)	+	281.36
	SSA	84.39 (0.53)	+	96.67 (0.71)	+	287.33 (9.16)	+	275.93
	LPSO	87.18 (0.63)	+	97.19 (0.61)	+	234.7 (8.29)	+	267.64
	HPSO-DE	94.05 (0.53)	+	93.84 (0.77)	+	35.57 (4.65)	+	224.65
	APSOLL	97.71 (0.33)	*∗*	98.07 (0.53)	*∗*	20.25 (1.23)	*∗*	228.91

PD	GWO	85.54 (2.25)	+	80.88 (3.54)	=	27.00 (9.84)	+	187.67
	PSO	71.54 (1.62)	+	74.60 (2.11)	+	268.4 (11.07)	+	185.17
	HHO	86.78 (1.28)	+	81.60 (1.90)	+	8.43 (6.53)	=	127.75
	FPA	68.77 (1.31)	+	74.60 (2.24)	+	338.03 (12.81)	+	174.27
	SSA	68.59 (1.44)	+	74.23 (1.99)	+	336 (13.24)	+	173.38
	LPSO	71.38 (1.96)	+	74.48 (2.60)	+	270.43 (14.95)	+	185.49
	HPSO-DE	86.88 (0.87)	+	83.26 (1.39)	=	35.20 (4.53)	+	197.66
	APSOLL	88.44 (0.85)	*∗*	83.92 (1.24)	*∗*	7.58 (2.22)	*∗*	152.82

CNAE-9	GWO	86.28 (1.22)	+	88.80 (1.53)	−	167.83 (20.93)	+	203.26
	PSO	77.41 (1.75)	+	88.25 (2.47)	−	409.80 (14.03)	+	197.09
	HHO	74.04 (1.83)	+	79.55 (4.12)	+	332.23 (80.68)	+	269.84
	FPA	73.91 (1.36)	+	83.79 (1.99)	+	420.70 (15.22)	+	185.77
	SSA	73.74 (1.85)	+	83.80 (2.51)	+	425.57 (12.44)	+	183.18
	LPSO	77.69 (1.27)	+	88.79 (1.92)	−	412.63 (14.06)	+	195.26
	HPSO-DE	69.52 (1.87)	+	77.60 (2.43)	+	422.40 (13.83)	+	200.46
	APSOLL	87.35 (0.55)	*∗*	85.03 (0.93)	*∗*	61.83 (5.38)	*∗*	210.71

QSAR	GWO	92.45 (0.54)	+	93.21 (0.66)	=	95.57 (9.18)	+	236.35
	PSO	82.20 (0.62)	+	92.01 (0.68)	+	416.70 (17.62)	+	327.26
	HHO	92.68 (0.45)	+	90.35 (0.83)	+	19.10 (12.23)	−	227.42
	FPA	80.13 (0.49)	+	91.16 (0.80)	+	466.93 (10.49)	+	323.93
	SSA	80.06 (0.48)	+	91.37 (0.71)	+	474.40 (11.67)	+	320.06
	LPSO	82.40 (0.55)	+	92.02 (0.59)	+	410.13 (14.90)	+	323.23
	HPSO-DE	92.44 (0.27)	+	91.14 (0.41)	+	46.30 (6.15)	+	207.01
	APSOLL	94.10 (0.55)	*∗*	93.11 (0.74)	*∗*	36.83 (7.84)	*∗*	231.28

**Table 5:** The optimal classification accuracy, number of selected features, and CPU time in comparison to traditional methods.

Datasets		ANOVA	CHI2	Pearson	Spearman	Kendall	MI	APSOLL
MIC	Acc (%)	90.39	94.31	90.39	90.39	90.39	90.39	92.55
	#F	31	6	29	56	59	19	2
	Time	3.38	3.24	4.03	13.04	8.15	164.07	123.69

Urban	Acc (%)	83.01	83.66	63.40	63.40	63.40	82.35	85.62
	#F	22	62	3	3	3	31	4
	Time	2.12	3.36	2.96	15.32	8.61	161.59	91.62

SCADI	Acc (%)	95.24	95.24	85.71	90.47	85.71	100	100
	#F	23	34	94	118	107	46	7
	Time	0.79	2.19	2.02	19.56	12.49	143.46	38.08

Arrhythmia	Acc (%)	63.97	63.24	63.24	63.97	63.24	63.97	75.74
	#F	55	12	8	17	13	4	6
	Time	2.94	2.34	8.09	56.24	40.11	675.94	127.73

Madelon	Acc (%)	89.87	89.36	71.41	71.41	71.41	79.36	91.15
	#F	17	13	499	499	499	48	17
	Time	38.03	40.01	49.72	223.64	137.44	2833.54	169.32

Isolet5	Acc (%)	86.97	85.47	84.83	85.26	85.26	87.82	92.08
	#F	245	289	378	351	223	204	46
	Time	62.04	61.61	57.49	561.71	197.98	8484.77	218.44

MF	Acc (%)	98.33	98.83	94.83	94.00	94.33	93.83	98.50
	#F	622	402	482	386	411	629	21
	Time	43.79	44.31	50.45	312.71	188.62	5533.33	105.40

PD	Acc (%)	79.30	87.67	80.18	81.06	81.06	78.41	85.90
	#F	4	140	16	24	25	70	9
	Time	61.75	62.94	62.16	395.49	234.74	2488.38	154.63

CNAE-9	Acc (%)	89.81	88.27	85.49	85.49	85.49	89.51	85.80
	#F	213	142	855	855	855	647	64
	Time	66.91	84.32	63.66	462.64	237.18	7087.23	221.19

QSAR	Acc (%)	91.52	91.72	91.72	91.72	91.72	91.72	93.88
	#F	110	105	984	984	984	461	33
	Time	126.37	135.70	125.28	1058.36	350.85	8411.24	221.18

## Data Availability

The data used to support the findings of this study are openly available in the UCI archive.
